# Nanoparticles as Theranostic Vehicles in Experimental and Clinical Applications—Focus on Prostate and Breast Cancer

**DOI:** 10.3390/ijms18051102

**Published:** 2017-05-20

**Authors:** Jörgen Elgqvist

**Affiliations:** 1 Department of Medical Physics and Biomedical Engineering, Sahlgrenska University Hospital, 413 45 Gothenburg, Sweden; jorgen.elgqvist@vgregion.se; 2 Department of Physics, University of Gothenburg, 412 96 Gothenburg, Sweden; jorgen.elgqvist@gu.se

**Keywords:** nanoparticles, theranostics, nanomedicine, prostate cancer, breast cancer

## Abstract

Prostate and breast cancer are the second most and most commonly diagnosed cancer in men and women worldwide, respectively. The American Cancer Society estimates that during 2016 in the USA around 430,000 individuals were diagnosed with one of these two types of cancers, and approximately 15% of them will die from the disease. In Europe, the rate of incidences and deaths are similar to those in the USA. Several different more or less successful diagnostic and therapeutic approaches have been developed and evaluated in order to tackle this issue and thereby decrease the death rates. By using nanoparticles as vehicles carrying both diagnostic and therapeutic molecular entities, individualized targeted theranostic nanomedicine has emerged as a promising option to increase the sensitivity and the specificity during diagnosis, as well as the likelihood of survival or prolonged survival after therapy. This article presents and discusses important and promising different kinds of nanoparticles, as well as imaging and therapy options, suitable for theranostic applications. The presentation of different nanoparticles and theranostic applications is quite general, but there is a special focus on prostate cancer. Some references and aspects regarding breast cancer are however also presented and discussed. Finally, the prostate cancer case is presented in more detail regarding diagnosis, staging, recurrence, metastases, and treatment options available today, followed by possible ways to move forward applying theranostics for both prostate and breast cancer based on promising experiments performed until today.

## 1. Introduction

In order to be able to combine both a therapeutic and a diagnostic function into a single molecular entity, the research on and development of theranostic nanoparticles (TNPs) have increased continuously during the last couple of years (the MeSH (Medical Subject Headings) term “theranostics” gave 0 hits for 2004 but 801 for 2016 on Pubmed) [1,2,3,4,5,6,7,8]. These type of nanosized drug vehicles have been developed and tested in different settings such as, for example, iron oxide, gadolinium, gold, manganese, or polymeric nanoparticles (NPs), quantum dots, and liposomes for the diagnosis and treatment of various diseases [1,2,3,4,5,6,7,8,9,10,11,12,13,14,15,16,17,18]. The dimensions of these different molecular nanostructures is generally less than 100 nm, and two of the common goals for all different settings is to maximize the drug loading capacity, and increase the specificity of the TNPs towards cancer cells [1]. The key benefit of TNPs is that they have the possibility to increase the therapeutic efficacy, partly due to ameliorated drug circulation times increasing the tumor uptake, but also to lessen the risk of unwanted toxic effects on healthy tissue [16,17,18,19,20]. Another important feature of the TNPs is that the diagnostic and therapeutic functionality could be localized to the exact same position due to the attachment of both these agents on the same molecular drug vehicle. Compared to other larger molecular vehicles these small NPs has a larger surface-area-to-volume ratio. This feature allows the TNPs to reach the capillary bed while at the same time carry a large variety of therapeutic and diagnostic agents themselves.

When considering the use of NPs for cancer treatment or diagnosis, the enhanced permeability and retention (EPR) effect plays a central role, although this effect seems to vary among individuals in the human population [2,19,21]. The EPR effect is also believed not to be present or even similar for all types of tumors, as well as not being the only parameter responsible for the efficacy of a certain NP application [22]. Parameters such as the level of unwanted drug release into the systemic circulation and the intra-tumoral allocation as well as the amount and kinetics of the intra-tumoral drug release will also determine the final efficacy [22].

However, the EPR effect can make TNPs accumulate, and be retained, to a higher degree in tumor tissue as compared to normal tissue, due to leakiness of the increased vasculature in the tumor tissue [19,20,23]. Magnetic resonance imaging (MRI) is a valuable tool for image and evaluate the degree of accumulation due to its high spatial resolution in both tumors and healthy tissue [24]. During MRI, Gd(III) (Gd^3+^) based contrast agents are very effective and highly paramagnetic substances, enhancing the contrast by increasing the T1 relaxation rate R1 (=1/T1), due to its rare electronic configuration of seven unpaired electron spins. By combining Gd(III) with different kind of NPs it would be possible to increase the accumulation and retention time in tumors, and therefore even more increase the MRI contrast [25,26,27,28,29,30,31,32,33,34]. TNPs could then be developed with the Gd(III)-based MRI contrast agent combined with, for example, a therapeutic drug for cancer such as gemcitabine [35,36]. 

Another strong argument for developing a NP based anti-cancer technology is that it could enable earlier detection of the disease. Early detection is most often absolutely crucial and decides whether a cancer patient will have the possibility to be cured, or just receive a treatment extending survival before finally succumbing to the disease. It is a well-known fact that the major cause of mortality in cancer is due to tumor metastasis [37,38,39]. In most cases, the dissemination of the cancerous disease is caused by tumor cells that have shed from the primary tumor and enters into the systemic circulation, i.e., circulating tumor cells CTCs. The idea of CTCs was first noticed by Dr. Tomas Ashworth already in 1869 [40]. However, while CTCs can only be confirmed and monitored in patients having a more or less advanced cancerous disease [39,41,42,43,44,45,46,47,48,49,50,51,52,53,54], the goal for a nanomedical approach would be to detect the CTCs at a much earlier time point compared to what is possible today [55,56].

The significance of the TNP technology regarding cancer in general is that it potentially could diagnose better, using multimodal imaging, and at the same time more effectively treat these diseases, especially in the disseminated cases. To achieve this, different types of isotopes could be attached to the TNPs. By attaching, for example, specific monoclonal antibodies (mAbs), fraction of mAbs, or peptides to these multifunctional TNPs highly specific and therefore targeted imaging and radiation therapies could be achieved. Personalized medicine might be possible using TNPs, since the imaging of drug accumulation in individual patients would be possible. Such imaging would refer to both tumor as well as healthy tissue and would then make it possible to estimate and predict, to a better degree than what is possible today, the therapeutic efficacy, and also make possible adjustments of ongoing regimens [2,19,20,21,23].

This article presents different type of NPs, imaging, and therapy options, as well as promising theranostic applications utilizing those techniques, with a focus on prostate cancer (PCa) and breast cancer (BC). Relevant PCa and BC references are presented and discussed in every section, but towards the end of the article the PCa case is given extra focus regarding diagnosis, staging, recurrence, metastases, and treatment options available today. Finally, possible ways to move forward applying theranostics for both PCa and BC are suggested and discussed based on promising experiments performed until today.

## 2. Nanoparticles for Prostate and Breast Cancer

The development of different and innovative NPs for various medical applications has increased tremendously during the past couple of years. Despite some problems with relatively low tumor uptake in some cancer applications, there is today a number of promising alternatives that have been tested or are under the development [57,58,59,60,61,62,63,64,65,66,67,68,69,70,71,72,73,74,75,76,77,78,79,80,81,82,83,84,85,86,87,88,89,90]. Below follows a presentation of important and interesting NP alternatives suitable for PCa and BC, some of which already have been tested for these type of diseases while some are still to be explored more thoroughly. For example, since the Nobel Prize in 2010 to Dr. Geim and Dr. Novoselov for their pioneering work on the two-dimensional material graphene, several promising NPs have since been suggested, developed, and tested based on that material. A prerequisite although, before any translation into clinical use of any NPs, is a meticulous survey of their pharmacological and toxicological properties. The presentation below summarizes the most important general characteristics of the NP options, and to what extent they have been used in the PCa and BC context so far. When possible, theranostic applications are referenced to and discussed shortly for each type of NP. In addition to theranostic applications, some studies in which only an imaging or therapeutic approach has been used are also referenced to for each type of NP.

### 2.1. Iron Oxide Nanoparticles

**Figure 1:**
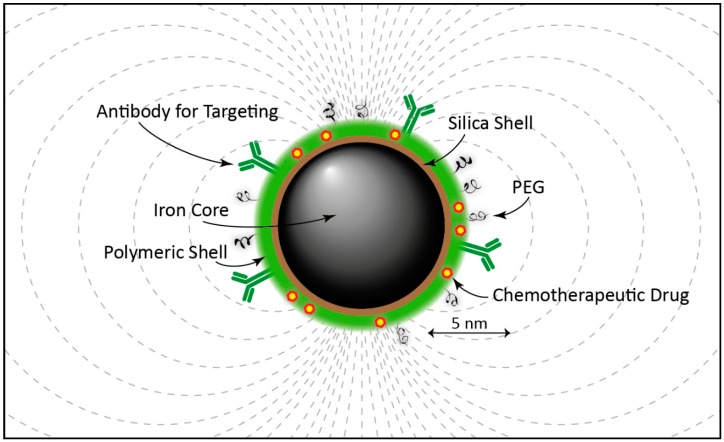
Functionalized iron-oxide nanoparticle. Functional biocompatible polymers are grafted onto an inorganic core of magnetite (Fe_3_O_4_) or maghemite (Fe_2_O_3_) through an anchoring group, such as an amine, carboxylic acid or phosphonic acid group. The polymeric shell improves the stability of the iron oxide nanoparticles (NPs) in solution, and also allows the encapsulation of, for example, therapeutic agents. An alternative coating in the form of a fluorescent silica (SiO_2_) produces a type of iron oxide NPs often referred to as SCIONs, i.e., silica coated iron oxide NPs. It should be noted that the polymeric shell has to be very opaque in order not to block the fluorescent silica-based core, if used simultaneous. Most commonly, experiments with only a polymeric or a silica core have been evaluated. The custom size of an iron oxide NP is in the range of 10–20 nm, as exemplified by a 10 nm in diameter iron core in the figure. Indicated in the figure are also the magnetic field lines created by this type of NP.

Due to their magnetic properties, with a diameter ranging from a few nanometers up to approximately 100 nm, iron oxide particles have been evaluated and used in several magnetic resonance technology-based biomedical applications such as multifunctional theranostic complexes combining tumor targeting, imaging as well as cancer nanotherapy in personalized cancer treatment [91,92,93,94,95,96,97,98,99,100]. The superparamagnetic iron oxide NPs (SPIONs) are most often used, and the subpopulation of ultrasmall SPIONs denoted as USPIOs defined as having a diameter of less than ~20 nm. A schematic representation of an iron oxide NP is shown in Figure 1. Three main variants of this NP are magnetite (Fe_3_O_4_), hematite (α-Fe_2_O_3_), and maghemite (γ-Fe_2_O_3_). The two latter being differently structured (rhombohedral and cubic, respectively) allotropic oxidized forms of magnetite. γ-Fe_2_O_3_ and Fe_3_O_4_ are preferred in medical applications due to their lack of toxicity and good biocompatibility in humans. Since SPIONSs tend to aggregate due to magnetic, van der Waals, and/or hydrophilic/hydrophobic forces it is important to minimize this effect in biomedical applications by different kind of surface modifications, for example, by PEGylation. PEGylation of a NP means attaching polyethylene glycol (PEG) molecules to its surface, thereby not only hindering aggregation but also masking the NP from the immune system. Other type of coatings can also be necessary for certain applications. For example, if SPIONs is used as a contrast agent during photoacoustic imaging, using near-infrared (IR) light, the NPs could be coated with silica (SiO_2_), enhancing the light absorption compared to the bare iron oxide NP [101,102,103]. Regarding diagnosis iron oxide NPs have been used for atherosclerotic evaluation, gene expression analysis, inflammation, angiogenesis, stem-cell tracking and also for cancer diagnosis.

Specifically, for PCa and BC there is ongoing research on different applications using these kind of NPs [93,104,105,106,107,108,109,110,111,112,113,114,115]. For example, regarding PCa, Zhu et al. have performed synthesis, characterization, an in vitro binding assay, and an in vivo magnetic resonance imaging (MRI) evaluation of prostate specific membrane antigen (PSMA) targeting SPIONs. They showed specific uptake of their polypeptide-based SPIONs by the PSMA expressing cells, and that the MRI signal could be specifically enhanced. They concluded that PSMA-targeting SPIONs might provide a new strategy for imaging PCa [107]. As an example regarding BC, Pasha Shaik et al. recently performed experiments on blocking the IL4-α receptor (IL4Rα) using PEGylated SPIONs to inhibit BC cell proliferation [111]. They found that for 4T1 cells, blocking of this receptor caused a significant decrease in cell viability and induced apoptosis. They also concluded that a combined treatment using SPION-IL4Rα-doxorubicin caused significant increases in cell death, apoptosis, and oxidative stress, compared to either SPION-IL4Rα or doxorubicin alone.

### 2.2. Gadolinium, Manganese, Gold, Silver, and Platinum Nanoparticles

For many years, gadolinium (Gd^3+^, Gd(III)) has been used in different contrast media for magnetic resonance imaging (MRI) during, for example, angiography or brain tumor imaging due to its paramagnetic characteristics, ability to shorten the T1 relaxation time, and to cross a degraded blood-brain barrier [116]. The research on contrast media based on the paramagnetic element manganese (Mn^2+^, Mn(II)) intended for MRI and NPs has just started to accelerate in recent years. For example, carbon and Mn^2+^ based NPs have been evaluated as contrast agents for MRI, or as manganese enhance MRI (MEMRI) during in vivo studies or for functional brain imaging [117,118,119,120,121,122]. Different applications such as Mn(II)–Au NPs as MRI contrast agents in stem cell labeling or Mn(II) based prussian blue(Fe_7_(CN)_18_)-based NPs as a theranostic agent having ultrahigh pH-responsive longitudinal relaxivity have also been investigated [123,124].

**Figure 2:**
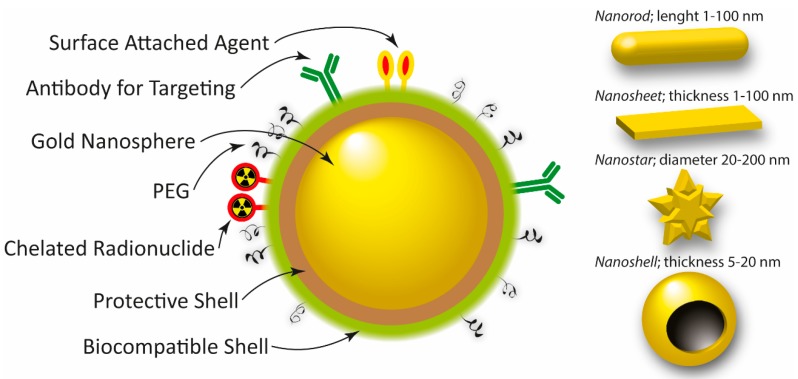
Image showing an example of a nanosphere of gold (Au). Gold nanoparticles (NPs) can also be based on, for example, nano-rods, -sheets, -stars, -or nanoshells, as indicated to the right with typical dimensions. The Au-nanospheres are most often produced in the size interval of 5 to 400 nm in diameter, approximately. The characteristics of AuNPs, and therefore their application possibilities, strongly depend on size, shape, and surface functionalities. Indicated in the image are also a protective and a biocompatible shell encapsulating the Au nanosphere, enabling the attachment of different kind of targeting vectors, imaging agents, therapeutic drugs, as well as polyethylene glycol (PEG) molecules. The latter an important parameter in order to protect the NPs from the immune system, to avoid reticuloendothelial system (RES) uptake, and to minimize nonspecific binding.

Gold NPs (AuNPs) in the form of nano-cages, -spheres, -beacons, -stars, -shells, -seeds, -sheets, or nanorods is being evaluated in many different settings, for example, for both PCa and BC (Figure 2) [8,125,126,127,128,129,130,131,132,133,134,135,136,137,138,139,140,141]. By changing the AuNPs’ shape, size, or surface characteristics it is possible to fine-tune their properties in order to maximize their applicability as a tool for cancer diagnosis, photo-dynamic/thermal therapy, therapy-drug carrier, radiotherapy drug enhancer, targeted gene therapy, or as a combined theranostic nanovehicle [142,143,144,145,146,147,148,149,150,151,152,153,154,155]. Regarding the gene therapy approach, in which for example, small-interference RNA (siRNA) could be utilized in order to knock out specific gene expressions in cancer cells, it has received increased attention recent years. For example, Guo et al. have recently shown interesting results on the PCa cells PC-3 and LNCaP indicating that two of their investigated formulations with transferrin and folate-receptor targeting ligands respectively (AuNPs-PEG-Tf and AuNPs-PEI-FA) show potential as non-viral gene delivery vectors in the treatment of PCa [141]. The photo-dynamic/thermal technique has also shown some progress recent years for both PCa and BC [156,157,158,159,160,161,162,163,164,165]. For example, Oh et al. have shown promising PCa cell killing efficacy by using a 55 nm small icosahedral phage that was engineered to display a gold-binding peptide as well as a PCa cell-binding peptide and applying a 60 mW/cm^2^ light irradiation [156]. Mkandawire et al. have recently investigated an alternative way to treat BC cells inducing apoptosis by targeting their mitochondria using AuNPs during photothermal treatment [164]. Regarding tumor detection, surface enhanced Raman spectroscopy (SERS) can be used for in vivo applications, and some studies have been performed recent years investigating its applicability for PCa and BC [129,161,166,167,168,169,170]. Ramaya et al. investigated the overexpression of prostate specific antigen (PSA) in LNCaP cells by using tetraphenylethylene (TPE) appended organic fluorogens adsorbed on AuNPs. Indoline-based TPE-AuNPs were efficient recognizing PSA overexpressing LNCaP cells using SERS mapping. For BC for example, Butler et al. investigated MCF-7 (Michigan Cancer Foundation-7) cells incubated with 150 nm AuNPs and concluded that they were a good starting point for near-infrared (NIR) or infrared (IR) SERS analysis [168].

The use of silver NPs (AgNPs) for medical applications has not been as intense as that of AuNPs. Regarding PCa and BC, however, there have been some studies published for different applications during recent years [171,172,173,174,175,176,177,178,179,180,181,182]. For example, Wang et al. developed a Ag-hybridized-silica-NP-based electrochemical immunosensor for the sensing of PSA in human serum with promising results [172], and Swanner et al. investigated the radiosensitizing and cytotoxic effect on triple-negative BC using AgNPs with good results at doses that have little effect on nontumorigenic BC cells [178].

Platinum-based NPs (PtNPs) is also a technique with a relatively low number of publications for PCa and BC applications, and then most often in the context of AuNPs, FeONPs, or for immunosensor applications [183,184,185,186,187,188,189,190,191,192]. Cui et al. developed an immunoassay based on mesoporous PtNPs, evaluated its efficacy against the BC tumor markers CA125, CA153, and CEA, and concluded its high potential clinic value [185]. Spain et al. recently proposed an electrochemical immunosensor based on PtNPs conjugated to a recombinant scFv antibody for the detection of PSA during PCa diagnosis, and showed that picomolar PSA concentrations could be detected without the need for further PCR or nucleic-acid-sequence-based amplification (NASBA) techniques [188].

### 2.3. Quantum and Cornell Dots

The semi-conductor metal based NPs called quantum dots (QDs) have, due to their adjustable properties, been used and tested in many electronic applications such as LCD displays and solar cells [193,194]. However, lately they are also evaluated in cancer research and medical-imaging applications and are usually fabricated in sizes of approximately 2–7 nm in diameter [195,196,197]. Examples of such, from blood rapidly excreted NPs based on combinations of different semi-conductor and heavy metals, are CdSe, CdS, PbS ZnS, InP, and CdTe, having fluorescence emission spectra in the region of 450–950 nm depending mainly on size and which type of coating the QDs have for a certain application [198,199]. A common coating is polyethylene glycol (PEG), which has the effect of increasing the blood circulation time by decreasing the kidney clearance.

Due to concerns regarding the heavy metal involvement in the QDs alternatives have been developed. One such option is the silica based Cornell dots (C dots) [200], first invented by Uli Wiesner (Wiesner Group, Cornell University, USA). The spherically shaped C dots are constructed with a silica based core, in which fluorescent molecules (fluorophores) are embedded, surrounded by a PEGylated silica shell [201]. In order to turn these C dots into targeted cancer probes one possibility is then to label chains in the PEG molecule with peptides or (fraction of) monoclonal antibodies (mAbs) that are site specific for certain cancer cell receptors. If the cancer cell bound C dots are illuminated using a near-infrared light source they fluoresce and can then serve as, for example, optical guidance for surgeons. Besides this diagnostic and surgical tool the C dots can of course also be labeled with suitable anticancer drugs or radioactive isotopes, and therefore also serve as a nanovehicle for targeted cancer therapy. It should be noted that PEGylation of C dots causes them to be rapidly excreted via the kidneys, as opposed to the QDs, decreasing blood circulation times [202,203,204,205]. A first clinical trial performed at the Memorial Sloan Kettering Cancer Center (MSKCC) in New York, USA, investigated radioactive iodine-labeled 7 nm C dots on five metastatic melanoma patients regarding positron emission tomography (PET) traceability and toxicity. The results showed that, under the U.S. Food and Drug Administration’s (FDA’s) Investigational New Drug (IND) guidelines, these type of NPs were safe for the use in humans [204].

Regarding applications for PCa and BC there are some publications using QDs or C dots [86,206,207,208,209]. For example, Zhao et al. recently evaluated QDs in a Cerenkov-imaging PCa model. [210]. They developed three different near-infrared QDs and ^89^Zr dual-labeled NPs and demonstrated the applicability of such self-illuminating NPs for imaging of lymph nodes and PCa tumors. For BC, applications of QDs is exemplified with a paper by Bwatanglang et al., in which they present results after investigating folic-acid functionalized chitosan-encapsulated QDs [208]. They found both enhanced binding affinity and internalization of their NP platform for folate receptor-overexpressing MCF-7 and MDA-MB-231 cells, and therefore concluded it to be a promising candidate for theranostic applications.

### 2.4. Carbon Based Nanoparticles

**Figure 3:**
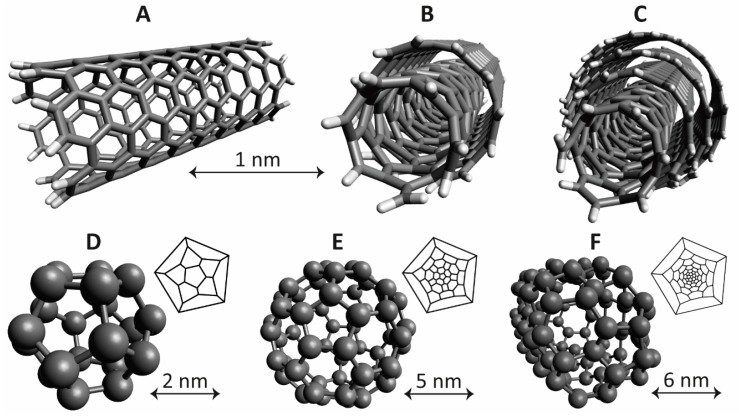
Examples of carbon-based nanotubes and fullerenes. (**A**) Single-walled carbon nanotube (SWCNT); (**B**) Double-walled carbon nanotube (DWCNT); (**C**) Multi-walled carbon nanotube (MWCNT); (**D**) Fullerene based on 20 carbon atoms (20-fullerene); (**E**) Fullerene based on 60 carbon atoms (60-fullerene); and (**F**) Fullerene based on 100 carbon atoms (100-fullerene). The top size indicator applies for panels **A**–**C**. The 3D-structures were created using Avogadro molecule editor. For panels **A**–**C** a rod-based representation, and for panels **D**–**F** a ball-based representation, was chosen for best clarity of the 3D-distribution of carbon atoms and the covalent bindings between them. For panels **D**–**F** is also shown 2D-representations of the fullerene structures, as well as individual size indicators. Note, the 100-fullerene has an obloid-like structure for its global energy minima, compared to the spherical 20- and 60-fullerenes, as discussed, for example, by Yoshida et al. [225].

The research on NPs based on carbon and allotropes of carbon such as fullerenes and graphene (e.g., nanohorns or nanotubes) have increased in recent years (Figure 3) [57,58,211,212,213,214,215]. These kind of NPs have received increased attention due to their chemical stability, favorable surface chemistry, high drug loading capacity, as well as high degree of variability. When it comes to medical applications attention has been particularly directed towards applications such as drug delivery, photothermal therapy, and imaging. Examples of this kind of NPs are fullerenes (spherical (i.e., buckyballs), ellipsoidal, or tube-shaped), carbon nanotubes (CNTs) such as single-walled CNTs (SWCNTs), double-walled CNTs (DWCNTs), or multi-walled CNTs (MWCNTs), carbon quantum dots (CQDs), graphene quantum dots (GQDs), and graphene oxide (GO). Regarding fullerenes they have been evaluated and utilized as for both X-ray and MRI imaging contrast agents, but also in applications for bringing a therapeutic substance to its target, such as gene delivery [216]. Different forms of CNTs can all be produced and chemically modified enabling labeling with, for example, radioactive isotopes [217,218]. Although promising applications of CNTs have been shown the question regarding toxicity of this nanovehicle is still under debate [219]. For example, it has been demonstrated that under certain circumstances the CNTs are able to cross the cell membranes of healthy tissue and induce harmful inflammatory and fibrotic responses, as well as cell death [220,221,222,223,224]. It should be noted though, that elevated risks are especially connected to chronic exposure to CNTs, which is not the case for medical applications for which the administration is performed under a limited period of time.

Regarding PCa and BC applications utilizing carbon-based NPs there is an increasing number of publications during the last couple of years, both for imaging and therapy but also for different kind of electrochemical biosensor systems [226,227,228,229,230,231,232,233,234]. For instance, regarding PCa, Heydari-Bafrooei et al. and Pan et al. have both developed different kind of CNT-based biosensor systems able to detect prostate specific antigen (PSA) in serum and vascular endothelial growth factor (VEGF) and PSA in serum, respectively, for early diagnosis of PCa [227,229]. For example, regarding BC, Misra et al. developed a carbon NP-DNA complex (CNPLex) used to transfect green fluorescent protein (GFP) reporter gene containing plasmid DNA (pDNA) pEGFP-N1 targeting BC cells MCF-7 and MDA-MB231 with promising results [234].

### 2.5. Liposomes

**Figure 4:**
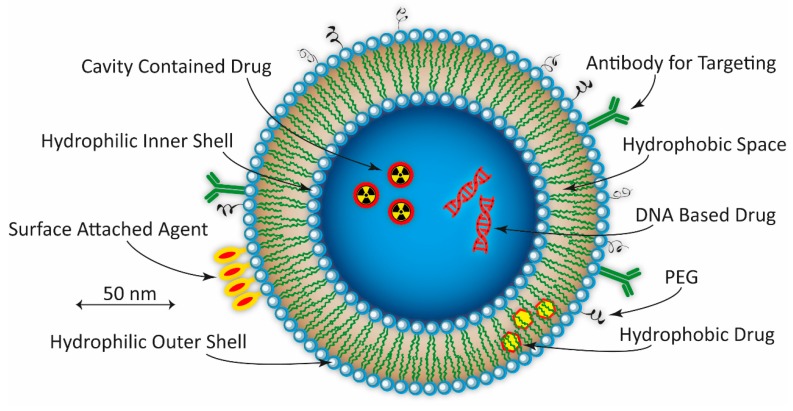
Schematic presentation of a spherical PEGylated liposome. Indicated are the different available spaces and surfaces that could carry different kind of targeting molecules as well as therapeutic drugs and imaging agents. In this hypothetical example, an antibody chelated to the outer surface is used for targeting. On that surface is also attached an imaging agent. Some kind of therapeutic drug is carried by the hydrophobic space between outer and inner shell. And in the core cavity, two hydrophilic drugs are situated, here exemplified by a radionuclide and a DNA-based drug such as strings of DNA, RNA, or small-interference RNA (siRNA). Unilamellar liposomes lies mostly in the size range of >150 nm, exemplified by a 200 nm liposome in the figure.

The phospholipid, principally phosphatidylcholine, based bilayer structure, constituting the body of the spherical vesicle called liposome, can be arranged in such a way as to produce a small unilamellar liposome vesicle (SUV) (Ø < 100 nm), large unilamellar vesicle (LUV) (Ø = 100–1000 nm), giant unilamellar vesicle (GUV) (Ø > 1000 nm), multilamellar vesicle (MLV), or a cylindrically shaped nanocochleate vesicle (NCV). An example of a hypothetical spherical unilamellar liposome is shown in Figure 4. The MLV’s are constructed by one or more unilamellar liposomes being encapsulated within a larger one. By disrupting the bilayer structure by ultra-sonication the liposomes can be prepared and loaded with, for example, different pharmaceutical drugs, either hydrophobic or hydrophilic. In such a way, the liposomes can act as vehicles for drugs directed against different diseases. The persistent and important work over several decades by the biophysicist Alec D Bengham on liposomes paved the way for its application as NPs in current biomedical research [235]. Depending on which type of liposome under consideration, the size ranges from around 20–100 nm (SUV), 100–1000 nm (LUV), to over 1000 nm for the MLV, and up to 200 µm for the GUV. The negative charges of the hydrophilic phospholipid heads on the surface of the liposome could be utilized for binding positively charged molecules and/or radioisotopes by electrostatic interaction [236]. Also, by specifically blocking these negatively charged heads using PEGylation, it is possible to increase the blood circulation time by minimizing the kidney clearance rate [237]. 

Studies utilizing liposomal-based NPs for PCa and BC theranostic techniques are few [238,239,240,241,242,243,244,245,246,247], although applications for therapy or imaging alone are significantly more frequently published [248,249,250,251,252,253,254,255,256,257,258,259,260,261]. For example, Yeh et al. recently published results from experiments investigating peptide-conjugated liposomal NPs in a theranostic approach for PCa. They found that the administration of liposomal doxorubicin and vinorelbine conjugated with targeting peptides increased the inhibition human PCa growth, and concluded that the targeting peptide SP204 has significant potential for both targeted imaging and therapy of PCa [238]. A liposomal-based theranostic approach against BC could be exemplified by the work by Rizzitelli et al. in which they evaluated the release of doxorubicin from liposomes monitored by MRI and triggered by ultrasound stimuli. The treatment led to a complete tumor regression in their BC mouse model [241]. 

### 2.6. Polymer Based Nanoparticles

**Figure 5:**
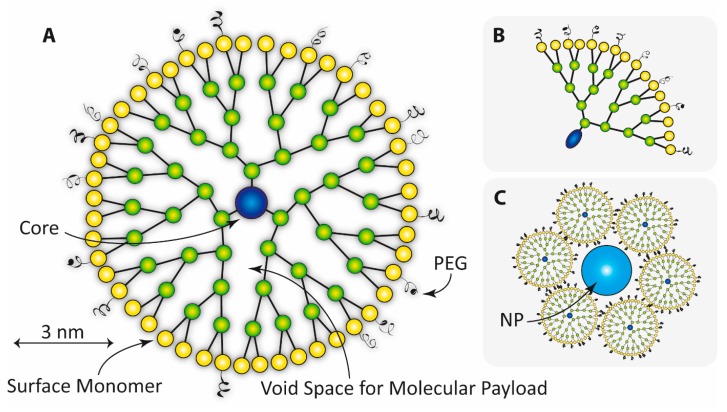
Schematic presentation of one type of spherical PEGylated dendrimer based on a central core (**A**), which in itself can be a dendrimer or some other type of NP (i.e., if so, a NP-cored dendrimer (NPCD)). A standard size of a dendrimer shown in A is 5–10 nm, exemplified by a 10 nm dendrimer in the figure. The surface monomers enable attachment of imaging and/or drug payloads, also able to be entrapped in the void space. The network of covalently bound interior monomers connects the surface monomers to the core. The number of radially emerging branch points defines the generation of the dendrimer; in this case four, denoted G-4. If instead, NPs are situated inside the network of monomers the term used is dendrimer-encapsulated nanoparticles (DENPs). Hyper-branched polymers are a variant similar to dendrimers, except that the branches emanating from the core differ from each other with regard to number of branch points. To the right is shown an example of a dendron (**B**), i.e., a small fragment of a dendrimer that also could be used as a NP. An example of a dendrimer-stabilized nanoparticle (DSNP) is shown in the lower right corner (**C**). Depending on if the functionalization is located on the surface, in the void space, or at the core of the dendrimer it is usually denoted as surface, interior, or core functionalized, or as a combination of all three possibilities.

These type of biodegradable block-copolymer based NPs can encapsulate and carry relatively large pharmaceutical molecules such as proteins, individual genes, or pieces of DNA [262]. Examples of polymers used for these types of NPs are polycyanoacrylate (PCA), poly d,l-glycolide (PLG), polylactic acid (PLA), polylactide-*co*-glycolide (PLGA), poly(isohexyl cyanoacrylate) (PIHCA), or polybutyl cyanoacrylate (PBCA) [262]. Among these polymers, PBCA and PIHCA have shown to be the fastest regarding biodegradability. For example, 24 h post an intravenous injection of PBCA it showed a level of reduction in the order of 80% [263]. PIHCA is currently undergoing clinical trials in phase III for hepatocellular carcinomas using the drug doxorubicin (Livatag^®^ (Doxorubicin Transdrug™), Paris, France). Three main NP structures, or micelles, can be achieved using polymers, namely nanocapsules, nanospheres, and nanoparticles. In the first case the pharmaceutical is encapsulated and completely surrounded by a spherical or rod-shaped shell of block-copolymers. In the second case, the pharmaceutical is embedded in a polymeric spherically shaped matrix. In the last case, the pharmaceutical is attached on the surface of the polymer based nanostructure [262]. The encapsulated, embedded, or attached pharmaceutical can of course be a radioactive agent of some sort [264]. Covalent pegylation of polymeric NPs can increase blood circulation times as well as facilitate uptake of the drug at targets aimed for [265]. Dendrons and symmetrical dendrimers (Figure 5) are a type of branched polymer based macromolecule that could be used as NPs [266,267]. Dendrimers have attracted much attention regarding theranostic applications, and the subject could easily fill a review on its own [268,269,270]. For example, hyperbranched PAMAM (polyamidoamine) dendrimer, based on hydrophilic ethylene diamine and investigated for medical purposes, can be labelled with monoclonal antibodies due to suitable amino groups in its structure [271]. The PAMAMs can be produced with multifunctional terminal surfaces and a narrow molecular weight distribution [272]. Finally, polymersomes is a type of polymeric NP for which amphiphilic synthetic block-copolymers are utilized to construct the membrane of the vesicle. Polymersomes have many similarities with liposomes, built by natural lipids (see above), but exhibit decreased permeability and increased stability compared to liposomes. The polymersomes have a size span of approximately 50–5000 nm.

There are very few theranostic polymer-based NP studies reported for both PCa and BC. In one such study Ling et al. evaluated multifunctional dual docetaxel/superparamagnetic iron oxide (SPIONs) loaded polymer vesicles (147 nm in diameter) for both imaging and therapy of PCa [273]. Enhanced cellular uptake and anti-proliferative effect for the PC3 cell line was observed which, in conjunction with the SPION-based MRI possibility, made the authors conclude that these polymer-based NP vesicles were promising for simultaneous imaging, drug delivery, and real-time monitoring of the therapeutic effect. For BC, a theranostic polymer based technique has been published by Abbasi et al. in which they experimented with Mn-oxide and docetaxel co-loaded fluorescent polymer-based NPs for dual-modal imaging and therapy of BC [274]. The authors concluded this type of polymer-based NP as a good candidate for cancer theranostic applications. Other interesting, however not yet fully theranostic, applications of polymer-based NP for PCa and BC have been published [275,276,277,278,279]. 

### 2.7. Solid Lipid Nanoparticles

**Figure 6:**
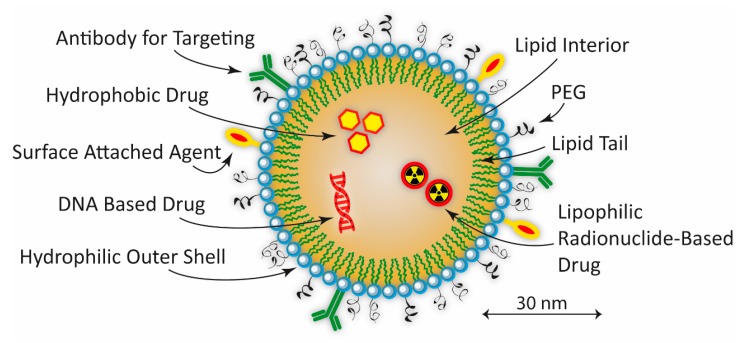
Solid lipid NP (SLNP). Although liquid lipid NPs are possible to produce, the most common form of lipid-based NPs investigated for medical purposes are SLNPs. The lipids most often used are fatty acids or different forms of glycerides. The smallest forms of SLNPs are in the shape of micelles, in which the fully dehydrated tails of the phosphatidylcholine molecules meet in the center, producing SLNPs in the size of 10 nm in diameter. Typical sizes for SLNPs are, however, most commonly in the interval of 5 to 500 nm in radii, exemplified by a hypothetical SLNP with a radius of approximately 30 nm in the figure.

The methodology of solid lipid NPs (SLNPs, or SLNs) is a promising emerging research field of lipid nanotechnology [280,281,282,283,284,285], which offers good possibilities to incorporate drugs into nanosized targeted vehicles having great biotolerability and low biotoxicity due to their constitution of physiological lipids [286]. Examples of such lipids are mono-, di-, and tri-glycerides, steroids, and fatty acids. Among the advantages of SLNPs could be mentioned the possibility of incorporating both hydro- and lipophilic drugs, good drug stability, and the lack of organic solvents in its composition [286]. The size of the SLNPs vary between 10 and 1000 nm, and the most common geometrical shape is spherical (Figure 6). The core is composed of solid lipids which is stabilized by emulsifiers, which also has the effect of decreasing the risk of NP agglomeration [286,287]. 

Until today, no fully theranostic application has been reported for neither PCa nor BC utilizing SLNPs. However, some studies have been published investigating the possibility using this NP system for either imaging or therapy alone [288,289,290,291,292,293,294,295,296,297,298]. For example, Radaic et al. investigated the possibility of gene therapy using SLNPs and tested the capacity of their NPs to accommodate DNA (and withstand DNase degradation), their colloidal stability and in vitro cytotoxicity, as well as the transfection efficiency in PCa cells [288]. For BC, Jain et al. recently published results investigating the anticancer efficacy of lycopene loaded SLNPs [294]. They found that the concentration and time dependent cell survival of MCF-7 BC cells was significantly reduced by LYC-SLNPs, as compared to their free lycopene counterparts.

## 3. Multimodal Imaging Options for Prostate and Breast Cancer

The use of various biomedical imaging techniques in preclinical and clinical applications, during both diagnosis and treatment has increased tremendously during the past decades, and is now considered a central part in many of such applications. Computed tomography, positron emission tomography, single photon emission computed tomography, magnetic resonance imaging, ultrasound imaging, Cherenkov luminescence imaging, photoacoustic imaging, and optical imaging are examples of images techniques used, and still under development. The images these different technologies produce enable early detection, screening, image-guided treatment, as well as the possibility of estimating the level of progression or retrogression of the disease investigated [299]. By using a NP-based targeted vehicle it is possible to use combinations of these imaging techniques simultaneously in order to increase the level of accuracy, possibly also at a cellular or even a molecular level. This NP-based multifunctionality in biomedical imaging could be referred to as multimodal imaging (MMI). Most often, only two imaging techniques is utilized simultaneously, which then is referred to as bimodal imaging (BMI). Below follows a condensed presentation of each of these different biomedical imaging techniques and their basic characteristics. When applicable, examples on how they have been used in NP-settings for PCa and BC applications so far, e.g., in MMI or BMI contexts, are shortly mentioned and referenced to.

### 3.1. Computed Tomography and Tomosynthesis

Regarding computed tomography (CT), contrast enhancers such as barium or iodine compounds (e.g., Gastrografin) could be used to increase the absorption of X-rays and thereby enhancing the contrast of tissues in an image, taking up these contrast agents. A targeted NP platform carrying such contrast enhancers could thus improve the anatomical visualization of organs and other structures, compared to non-targeted regions. But also some NPs themselves can improve the contrast, exemplified by AuNPs increasing the contrast approximately three times as compared to the same amount of iodine [300]. Differently sized AuNPs have been evaluated for micro CT for example, and some are under the consideration for approval for use in the clinic [301,302,303]. A low-density lipoprotein (LDL)-based iodinated nanoparticle targeting the LDL receptor (LDLR), expressed in PCa, for example, has also been evaluated for CT imaging [304,305,306], as well as polyvinyl pyrrolidone coated bismuth sulfide (Bi_2_S_3_) based nanocrystals [307]. For tomosynthesis, the same general principle applies as described above for CT, namely that the contrast in an image could be increased approximately three times using AuNPs as compared to using the same amount of iodine-based contrast agent. For contrast-enhanced breast tomosynthesis (CE DBT), temporal or dual-energy subtraction techniques are of course still possible to use regardless of which type of contrast agent is utilized [308].

Most applications of CT in NP contexts are based on positron emission and single photon emission computed tomography (see below). However, one example in which CT has been used is described by Kim et al., in which they investigated a multifunctional gold-based NP system for both contrast-enhanced imaging and therapy of PCa [309]. RNA-aptamer functionalized gold NPs targeting PSMA enabled specific imaging of PCa. When also loaded with doxorubicin their theranostic NPs showed good therapeutic efficacy against LNCaP cells. For BC, a study performed by Naha et al. evaluated gold silver alloy NPs as an imaging probe for BC screening using conventional CT as well as dual-energy mammography (DEM) [310]. In vivo experiments in mice exhibited good tumor accumulation of the NPs and produced high contrast DEM and CT images, enabling the authors to conclude that their NP system has potential for both blood pool imaging and BC screening.

### 3.2. Positron Emission and Single Photon Emission Computed Tomography

Radio imaging using positron emission tomography (PET) and/or single photon emission computed tomography (SPECT) they have been evaluated for several NP applications using both organic and inorganic NPs, for example, in PET/CT or SPECT/CT settings [311,312,313,314]. Using a NP platform makes it possible to increase the contrast in an image due to the possibility to label each NP with a large number of radionuclides. For PET, examples of radionuclides used in such applications are ^18^F, ^124^I and ^64^Cu, and for SPECT examples are ^125^I and ^125^Cd.

Regarding PCa, PET (or PET/CT) and SPECT (or SPECT/CT) has been used in several NP applications [131,315,316,317,318,319,320,321]. For example, Pressley et al. evaluated an amphiphilic NP for natriuretic peptide clearance receptor (NPRC) targeting and DOTA (1,4,7,10-tetraazacyclododecane-1,4,7,10-tetraacetic acid) chelator for high specific activity ^64^Cu PET-radiolabeling. PET/CT images revealed high blood pool retention, low renal clearance, enhanced tumor uptake, and decreased hepatic burden relative to a nontargeted NP version [317], indicating the possibility of a new nanoagent for PCa PET imaging, according to the authors. In NP-based BC applications, PET and SPECT have been utilized in several studies [322,323,324,325,326]. For example, Lee et al. recently published a study in which they assessed the EPR effect in nineteen patients with HER2-positive metastatic BC using a ^64^Cu-labeled NP (^64^Cu-labeled HER2-targeted PEGylated liposomal doxorubicin) using PET/CT [322]. The authors concluded that the results provide evidence and quantification of the EPR effect in human metastatic BC tumors, as well as support NP-deposition imaging as a potential technique for identifying patients well-suited for NP-based therapeutics.

### 3.3. Magnetic Resonance Imaging

The magnetic resonance imaging (MRI), or magnetic resonance tomography (MRT), technology is today widely used for imaging physiological processes as well as the anatomy during both preclinical research and in the clinic [327]. Varieties of this technique includes functional MRI (fMRI), measuring levels of cerebral blood flow, as well as techniques to increase the contrast in MRI images such as dynamic contrast-enhanced MRI (DCE-MRI), utilizing a contrast agent such as gadolinium, and diffusion-weighted MRI (DW-MRI), utilizing the Brownian motion of water molecules. The MRI tool plays an important role in the staging of PCa (see below in section Prostate Cancer) and will most likely be applicable and show an increased importance also for other types of cancers, such as BC, for both diagnosis and staging, especially if used in a NP setting, discussed in this paper.

The ^19^F-based MRI [328,329,330,331,332] offers many advantages, despite the difficulty of providing suitable non-toxic ^19^F-based compounds in sufficient amounts for in vivo imaging, compared to common proton-based ^1^H-MRI such as decreased background levels, quantitative determination of pharmacokinetics, and estimation of tissue oxygenation [300,333,334]. The use of ^19^F-MRI in NP applications is a developing research field investigating, for example, the applicability compared to SPIONs, the efficacy of fluorinated dendrimers and multifunctional micelle-based core–shell NPs, and the detection of folate-receptor positive tumors in a ^19^F/florescence-based bimodal imaging setting [331,335,336,337]. Just recently, a ^19^F based nanoemulsion has been FDA approved for noninvasive clinical cell-tracking imaging [338].

Regarding MRI/MRT used in PCa related NP settings the number of publications is constantly increasing [104,339,340,341,342,343,344]. Jin et al., for example, evaluated MRI-guided focal NP-based (porphysomes) photothermal therapy (see below) in an orthotropic PCa mouse model, and concluded that it might be an effective and safe technique to treat PCa, with a low risk of progression of disease [343]. For BC, there is also a large number of publications [91,345,346,347,348,349]. In a study by Turino et al. l-ferritine-coated paclitaxel- and Gd-loaded NPs were evaluated for simultaneous delivery of a therapeutic drug and a MRI-contrast agent in a MCF7 BC model [345]. According to the authors, the theranostic potential of this NP system was demonstrated by, for example, evaluating signal-intensity enhancements in T1-weighted MRI images.

As for CT, by combining the MR-imaging mode with PET enables localization and biodistribution at the same time. Very few studies are however reported until today using this technique in NP settings for PCa and BC [350,351,352].

### 3.4. Ultrasound Imaging

During ultrasound imaging (USI), utilizing contrast agents to improve the contrast, particle sizes used often exceed 250 nm. Although the most commonly used size definition for NPs is 1–100 nm, the USI contrast agents is still appropriate to mention here since USI can play an important role in MMI. Two examples of commercially available USI contrast agents are Definity and Optison based on microbubbles. Both type of bubbles contain octafluoropropane gas, while Definity uses a phospholipid spherical shell and Optison an albumin based shell to enclose the gas. Experimentally, SPIONs, liposomes, AuNPs, and nanodroplets have also been evaluated as contrast agents and drug carriers during NP-based US applications [353,354,355,356,357,358]. All types of microbubbles can serve as platforms for not only imaging, but also for the distribution of drugs for therapy by encapsulating the drug into the bubbles [359,360]. Targeted microbubbles for use during both imaging and therapy, or at the same time as targeted theranostic vehicles, can be achieved by attaching ligands on the surface of the bubbles which specifically bind to receptors on, for example, tumor cells.

In the study by Tong et al. mentioned above for PCa, a protamine cationic microbubble (Ø ≈ 500 nm) was constructed for simultaneous gene therapy (androgen-receptor siRNA) and ultrasound imaging [360]. The authors concluded that the gene-transfection efficiency was better than that of a liposomes-based comparable system, and that their microbubble system could be used as a gene-loading and ultrasound-imaging technique of tumors. For BC, Zhao et al. recently investigated a near-infrared (808 nm) photothermal responsive dual aptamers-targeted docetaxel-containing NPs for both cancer therapy and USI [353]. The dual-ligand functionalization increased uptake in MCF-7 cells and made USI possible at tumor site. The authors therefore concluded their system to be a promising theranostic option involving light-thermal response, dual ligand targeted triplex therapy (chemotherapy, photothermal therapy, and biological therapy), and USI.

### 3.5. Cherenkov Luminescence Imaging

The Cherenkov luminescence imaging (CLI) technology, named after Pavel Alekseyevich Cherenkov who shared the Nobel Prize in physics in 1958 for the discovery of the now called Cherenkov effect, has during recent years emerged as an alternative imaging technique for several different applications [361,362,363,364,365,366,367]. The electromagnetic Cherenkov radiation, emitted when charged particles passes through a medium at a speed greater than light propagates through the same medium, could be used to image, for example, the uptake of a charged-particle emitter in tumors during radioimmunotherapy. There are very few publications on PCa and BC using CLI [368,369,370,371], among which very few have a NP approach. Lohrman et al. found a positive correlation between the radioactivity uptake and CLI signal from the ^90^Y labeled gastrin-releasing peptide-receptor (GRPR) antagonist DOTA-AR in xenografted PCa tumors [370]. Regarding using CLI in NP applications besides PCa and BC there are some publications [372,373,374,375]. For example, Madru et al. investigated the usability ^68^Ga-labeled SPIONs for multi-modality PET/MR/CLI imaging of sentinel lymph nodes (SLNs). Based on promising biodistribution experiments, the authors concluded that ^68^Ga-SPIONs can enhance the identification of SLNs by combining PET and MR imaging, and potential also enable Cherenkov luminescent-guided resection of SLNs [372].

### 3.6. Photoacoustic Imaging

A relatively new emerging imaging modality is photoacoustic imaging (PAI), although already with some PCa and BC orientated studies published the past decade [376,377,378,379,380,381,382,383]. PAI could be achieved by irradiating a biological site with a pulsed laser-beam in the megahertz range, which energy is then absorbed by, for example, targeted NPs. The absorbed energy creates acoustic pressure waves, caused by thermoelastic expansion, in the irradiated site. These waves could then be detected by using an ultrasonic transducer, enabling an image to be constructed. If instead of using a laser, pulses in radio-frequency range are used, the technique is called thermoacoustic imaging (TAI). Su et al. investigated recently mesoporous-silica coated and PEG modified multifunctional doxorubicin-loaded prussian-blue nanocubes (PB@mSiO_2_-PEG/DOX). Good both MRI and PAI ability, as well as a synergistic photothermal and chemical therapeutic efficacy, for BC was found [377]. For PCa, Levi et al. evaluated a PAI agent (AA3G-740) targeting the gastrin-releasing peptide receptor (GRPR), highly overexpressed in PCa [380]. The study showed that, even for poorly vascularized tumors, AA3G-740 was able to bind to GRPR and led to a significantly higher photoacoustic signal relative to a control agent.

Regarding NP applications using PAI there are some studies published for both PCa and BC [230,382,384,385,386,387,388,389,390,391]. By using PCa cells, Tian et al., for example, constructed PEGylated and RGD (Arginine-Glycine-Aspartic)-peptide functionalized AuNPs and evaluated them as a contrast agent for PAI of single PCa cells. The authors concluded that these NPs provide a platform for detection and imaging of individual cancer cells, with a potential impact on clinical diagnostic [384]. Pham et al. used mouse models based on orthotopic primary triple-negative BC xenografts (including patient-derived xenografts) to evaluate the efficacy of bevacizumab (a VEGF pathway-targeting antiangiogenic drug) in combination with CRLX101, an NP-drug conjugate containing camptothecin (a cytotoxic quinoline alkaloid inhibiting DNA topoisomerase). PAI was used in the study to show that the use of CRLX101 led to an improved tumor perfusion as well as reduced hypoxia. The authors concluded that pairing antiangiogenic therapy with a cytotoxic NP construct may be a promising way to treat metastatic BC [391].

### 3.7. Optical Imaging

The Optical imaging (OI) technology (sometimes referred to as biophotonics), the inclusive term often used for different types of infrared, ultraviolet, and visible light, as well as sometimes also photoacoustic (see above) applications in biomedical imaging, have a number of interesting publications regarding NP applications for both PCa and BC [319,392,393,394,395,396,397,398,399,400]. Generally, a NP-based OI approach could enable or optimize an optical excitation energy in, for example, tumor tissue, enable multispectral imaging by combining spectroscopy and OI, or make possible multiplex imaging by using different color emitters for different targets simultaneously [300]. For cancer applications, Ahir et al. recently developed a copper oxide-nanowire NP decorated with folic acid and studied its effect on triple negative BC (TNBC). They found that their NPs induced apoptosis and retarded migration of the TNBC cells, and used optical fluorescence imaging to monitor its distribution in tumors and different organs [392]. Regarding PCa, Behnam et al. constructed and investigated a PSMA-targeted bionized nanoferrite (BNF) NP in an experimental PCa model [319]. The study used near-infrared fluorescence microscopy, SPECT, Prussian blue staining, immunohistochemistry, and biodistribution to show an enhanced NP uptake in PSMA-positive tumors, with a maximum uptake 48 h post injection.

### 3.8. Electron Microscopy

Regarding electron microscopy (EM) in general, but especially transmission electron microscopy (TEM), it has an important role to play for the in vitro and ex vivo analyses due to its often sub-nanometer spatial resolution. Nanoparticles based on heavy elements such as gold could therefore be used for TEM applications in order to retrieve information on, for example, NP distribution on the organelle level. So far though, this technique has only been used occasionally in PCa and BC NP applications. Since EM is not considered to be an imaging technique possible to use in theranostic contexts, its value lies instead in in vitro and ex vivo analyses, as mentioned above, or during the production process of the NPs in order to be able to characterize them properly [401,402,403,404,405,406,407,408].

## 4. Multimodal Therapy Options for Prostate and Breast Cancer

Due to differences in metabolic and chemical stability, level of solubility in blood serum and interstitial fluid, degree of toxicity, and most important level of specificity for a certain tumor type as well as potency once properly targeted, several different drugs have been evaluated and some approved for targeted therapies against cancer [409,410]. For a detailed compilation of the NP-based technology and therapy of cancer in general the reader is referred to Professor P.N. Prasad’s fine textbook on the subject [300]. A NP-based therapeutic, in some cases also potentially synergistic, multifunctionality can be referred to as multimodal therapy (MMT), or if only two therapies are used simultaneously, as bimodal therapy (BMT). Below follows a presentation of different therapy options that all could be implemented in various NP settings. The basic characteristics and principles of each modality are presented briefly. References are also listed and some specific examples on how some of the available therapy modalities have been utilized in NP-settings for PCa and BC are discussed shortly, e.g., in MMT or BMT contexts.

### 4.1. Chemotherapy

Treatments using chemotherapy (CTH), since many years successfully applied and still under development for a wide category of cancers including PCa and BC, has limitations due its relatively high degree of non-specificity, inducing toxicity [411,412,413,414,415]. A targeted NP-based approach has shown to be beneficial and has been evaluated by many research teams. Some such NP-based CTH drugs have been FDA approved; the albumin-paclitaxel-based Abraxane^®^ and the PEG-doxorubicin-based Doxil^®^ for metastasized BC, the latter being the first FDA approved nanodrug [416,417]. But also other formulations are being evaluated in the clinic, or have already been approved or are being marketed in, for example, Europe. Such examples for BC is the non-pegylated liposomal-doxorubicin-based Myocet^®^ or the polymeric micelle-paclitaxel-based Genexol-PM^®^ [418,419]. An update from 2014 of FDA approved NP-based cancer drugs, and also others at various stages of development, has been published [420]. Regarding targeting of NP-based CTH drugs research are ongoing in order to, instead of relying on the passive targeting caused by the EPR effect, develop strategies for active targeting using, for example, mAbs (or fraction of mAbs) directed against the PSA receptor in the PCa case [421].

However, a large number of studies, using different techniques, have been published with a NP and CTH-based approach for both PCa and BC, of which only a tiny fraction are listed here [105,253,315,353,422,423,424,425,426,427,428,429,430,431]. For example, Belz et al. recently designed ultra-small silica NPs containing the radiosensitizing drug docetaxel for combined chemoradiation therapy, with potential benefit for patients with PCa [425]. For BC, Li et al. found synergistic inhibition of both migration and invasion of 4T1 BC cells by doubly loaded NPs (docetaxel + the Akt inhibitor quercetin), via the Akt/matrix metallopeptidase 9 (MMP-9) pathway [431].

### 4.2. Gene Therapy

The gene therapy (GTH) alternative has during the last two decades evolved as a promising tool for the treatment of cancer, either as a stand-alone therapy or in conjunction with chemotherapy, surgery, and/or radiation therapy [432,433]. The development of GTH towards treatment based on an individual’s specific genome, immune status, and tumor characteristics, together with new vectors for transferring the genetic material such as synthetic viruses as well as non-viral methods will further refine this still experimental, treatment option [434]. By adopting an NP-based GTH approach, the treatment is believed to be improved even further, especially when implemented as TNPs enabling imaging simultaneous with therapy. For the two main groups of genes associated with cancer, i.e., tumor-suppressor genes and oncogenes, examples of nucleic-acid based therapeutic molecules that are being evaluated for GTH are cytotoxic or corrective genes, small interfering RNA (siRNA) or short hairpin RNA (shRNA) [435].

Regarding NP-based GTH techniques for BC there are very few, however an increasing number, publications the last decade [234,436,437,438,439,440,441,442,443,444]. Su et al., for example, investigated recently the efficacy of a combinational technique including photothermal therapy (see below), CTH, and GTH for triple negative BC [442]. Indocyanine green, paclitaxel, and survivin siRNA was integrated into a NP and was found to exhibit very good tumor efficacy with low toxicity. The protein survivin, encoded by the *BIRC5* oncogene in the human genome, and which inhibits the caspase activation and therefore downregulates the apoptotic pathway, has received much attention lately. Several attempts, also including NPs, have been made to distribute anti-survivin siRNA in order to silence the BIRC5 gene [445,446,447]. For NP-based GTH applications for PCa, there are also quite few publications the last decade [141,448,449,450,451,452]. Guo et al. investigated, for example, gene silencing using siRNA-based AuNPs for LNCaP cells, overexpressing PSMA. With AuNPs conjugated with folate-receptor targeting ligands it was found that siRNA was specifically delivered into the LNCaP cells, and produced enhanced endogenous gene silencing [141].

### 4.3. Photon Activation Therapy

The photon activation therapy (PAT), sometimes also referred to as photon activated therapy, involving Auger electrons and mentioned for the first time for medical applications over three decades ago, shows a limited amount of publications but is an interesting option for TNPs and therefore discussed here [453,454,455,456,457,458,459,460,461]. The PAT technique is based on the principle of specific tumor localization of a high-Z compound such as platinum (Pt), incorporated in, for example, a CTH drug, after which synchrotron radiation or X-rays directed against the tumor site is used to, via the photoelectric effect, trigger a cascade release of high linear energy transfer (high-LET) Auger electrons. Except Pt, other nuclides investigated for PAT have been Au, Tl, Gd, I, and Fe. In the Pt case, the photon energy suitable for triggering this effect should be just above the binding energy of the K-shell electrons, i.e., 78.4 keV. As for α-particles, the mean LET value for Auger electrons is considerable higher than that of, for example, beta-particles; ~100, ~15, and ~0.2 keV/µm, respectively. This means that, as for α-particles, the Auger electrons will create densely ionization tracks causing damages in the cells, such as double strand breaks (DSB), which are very difficult to repair. An additional advantage with Auger electrons, compared to α-particles, is that their range in tissue is on the nanometer scale, compared to 50–100 µm for α-particles. So, provided that the Auger-electron emitting nuclide is being properly targeted in close proximity to, or incorporated into, the DNA of the tumor cells, a highly targeted high-LET irradiation will be achieved.

Regarding PCa and BC utilizing a NP-based PAT approach there is only one study published, having only a tentative BC relevance [462]. In that experiment Choi et al. investigated the therapeutic efficacy on colon cancer tumor-bearing mice injected with FeO NPs and irradiated using 7.1 keV synchrotron X-rays, an energy near the Fe K-shell binding energy. For example, one group that received FeO NPs and an absorbed tumor dose of 10 Gy showed 80% complete tumor regression after 15–35 days. As noted by the authors however, the use of 7.1 keV X-rays, having a high tissue attenuation, makes the treatment only suitable for superficial skin malignancies, and possibly also for superficial chest wall recurrence of BC.

### 4.4. Photodynamic Therapy

Photodynamic therapy (PDT), also referred to as photochemotherapy, utilizes a photosensitizing chemical substance called a photosensitizer (PS) that is irradiated with light at certain wavelengths to induce the production of molecular oxygen in the form of reactive oxygen species (ROS). The ROS, e.g., superoxide, peroxide, singlet oxygen, or hydroxyl radicals, have the capacity to induce cell death at the site of production and can therefore, if targeted properly, be used as a therapeutic option against several diseases [463]. Acne and psoriasis, or to some extent even herpes experimentally, are treated using PDT. But also different type of cancers, in particular different types of skin cancer, are being treated with PDT techniques [464]. Both wavelength and fluence of the light are important parameters to monitor in order to target and trigger the PS properly, using a laser-equipped endoscope as a special case for reaching, for example, intestinal cancers [465].

An interesting version of PDT is the two-photon excitation (TPE) based PDT for the treatment of cancer. This technique combine the advantages of TPE near-infrared (NIR) photosensitizers and nanotechnology and has been reviewed by Shen et al. [466]. The absorption of two relatively low-energy NIR photons will enable the emission of high-energy photons in the visible spectrum, which in its turn will sensitize oxygen producing singlet oxygen and reactive oxygen species (ROS) able to kill targeted cancer cells due to its cytotoxic effect. Compared to single-photon based PDT the possibility of reaching further into tissues, due to the relatively long wavelength of the light used in TPE PDT, has great advantages enabling to reach tumors more deep seated. There are some publications using the TPE technique, both for imaging and therapy, in different NP and theranostic settings [467,468,469,470,471]. The paper by Gary-Bobo et al. [471] was the first two-photon based PDT experiment in vivo using NPs.

Regarding PCa, PDT approaches have been evaluated both preclinically and for patients [472,473]. Especially, studies using PDT as a theranostic approach for PCa has lately also been published. For example, Chen et al. recently investigated a low-molecular-weight theranostic photosensitizer denoted YC-9 for PSMA-targeted optical imaging and PDT [474]. The study indicates that YC-9 is a promising therapeutic agent for targeted PDT of PSMA-expressing tissues, such as PCa. Similarly, Wang et al. synthesized two PSMA-targeting PDT conjugates (PSMA-1-Pc413 and PSMA-1-IR700), both having the potential to aid in the detection and resection of PCa [475]. Lin et al. developed a novel nano-platform for targeted delivery of heat, ROS, as well as the heat shock-protein 90 (Hsp90) inhibitor for the treatment of PCa [476]. Vaillant et al. investigated targeting a membrane lectin using a mannose-6-phosphate analogue grafted onto the surface of functionalized mesoporous silica NPOs [477].

For BC, several approaches have been evaluated. For example, Feng et al. investigated a multimodality theranostic agent based on mesoporous copper sulfide NPs encapsulating doxorubicin, enabling both PAI as well as chemo- and ROS generating phototherapy of BC [400]. Against TNBC, Choi et al. developed photosensitizer-conjugated and camptothecin-encapsulated hyaluronic acid NPs as enzyme-activatable theranostic NPs for near-infrared fluorescence imaging and photodynamic/chemo dual therapy [478]. Both in vitro and in vivo, Wang et al. performed experiments evaluating the effects of sinoporphyrin sodium-mediated PDT on tumor cell proliferation and metastasis for the highly metastatic 4T1 BC cells and a mouse xenograft model [479]. Targeting the TrkC (tropomyosin receptor kinase C) receptor, which tends to be overexpressed in metastatic BC, with a ROS photosensitizer-labeled small molecule enabled Kue et al. to investigate the therapeutic efficacy of PDT in nude mice [480]. Finally, Shemesh et al. used a liposomal-based theranostic delivery system, with indocyanine green as a photosensitizer, for investigating real-time biodistribution monitoring as well as the efficacy of PDT against TNBC [244].

### 4.5. Photothermal Therapy

The photothermal therapy (PTT) approach builds on the PDT principle (see above) in that it via passive (e.g., via the EPR effect) or active (e.g., via mAbs) tumor accumulation of nanoheaters/photosensitizers enables a localized temperature increase. This could cause the destruction of DNA/RNA molecules as well as proteins, leading to cell death by membrane rupture or necrosis [300,481]. The difference of PTT compared to PDT is that the former does not need oxygen present in order to induce cell killing. Especially one version of PTT has attracted increased attention, namely plasmonic PTT (PPTT) [482,483,484]. The PPTT technology is based on the principle that when AuNPs are irradiated using infrared or near-infrared light coherent excitation of its conduction electrons at the surface will take place, due to the surface plasmon resonance (SPR) effect. When these electrons deexcite, they will produce localized heat waves causing wanted cell destruction.

Regarding PCa and BC there are several publications investigating PTT in different NP settings [91,156,228,230,247,343,422,485,486]. For example, Hosoya et al. evaluated a theranostic hydrogel-based NP platform combining both targeting of the tumor cells, photon-to-heat conversion, as well as triggered drug delivery enabling controlled release of the anticancer drug and multimodal imaging [247]. Their results showed the possibility of simultaneous targeted delivery of an anticancer drug and noninvasive imaging for both PCa and BC in a mouse model. Also, Cantu et al. investigated polymeric NPs (<100 nm in diameter) in a photothermal ablation setting. When experimenting on MDA-MB-231 BC cells they were able to show complete cancer cell ablation in vitro using an 808-nm laser, indicating the potential benefit of their NP platform utilizing the PTT technology.

### 4.6. Radioimmunotherapy

The cancer treatment modality termed radioimmunotherapy (RIT) is since many years a well-established technique to specifically irradiate targeted tumor cells using monoclonal antibodies (mAbs), or fraction of mAbs, labeled with suitable radioactive isotopes such as α-, β-, or Auger-electron emitters. Review papers regarding the current RIT status for PCa and BC is referred to for further reading [487,488,489,490,491,492]. Regarding NP-based platforms for cancer utilizing RIT, sometimes referred to as radioimmunonanoparticles (RINPs), there is a small but increasing number of publications [493,494,495,496]. For PCa and BC, there have been only a few papers presenting some promising results [497,498,499,500,501]. Natarajan et al., for example, published a paper in 2008 presenting a potential theranostic approach in a PCa and BC experiment [501]. They developed a novel ^111^In-radioconjugate NP based on anti-MUC-1-scFv antibody fragments and functionalized NPs.

### 4.7. Neutron Capture Therapy

The radiation-based technique called neutron capture therapy (NCT) is based on a neutron source in order to generate a targeted internal radiation therapy at the specific tumor site, and has been described in several publications [300,502,503,504]. The technique is still a highly active research area, and applications in which it is evaluated now also includes theranostic NP-based settings [505]. Most applications so far have been exploiting the nuclear reaction ^10^B(n,α)^7^Li, i.e., bombarding boron atoms with thermal neutrons to produce internally emitted α-particles. This technique is called boron neutron capture therapy (BNCT). The isotope ^157^Gd has also been evaluated for NCT, although it has been questioned due to toxicity concerns regarding Gd^3+^ ions. However, chelation using DTPA has been promising and capable of producing stable Gd-DTPA complexes, and therefore nontoxic. The isotope ^157^Gd has some advantages over ^10^B, including, for example, a 67 times higher cross section for thermal neutrons as well as Gd^3+^ ions being paramagnetic and thereby able to function as contrast enhancers during MRI [300]. Regarding NP-based applications of NCT, it could help to increase the accumulation of ^10^B or ^157^Gd in the targeted tumor tissue. Liposome-based NP techniques is a possible approach and some experiments have shown promising results [504,506,507,508].

Regarding PCa and BC there are very few studies published using NP-based NCT techniques. Only two BC-related publications are to be found on PubMed, investigating dendrimer- and lipid-based gadolinium NPs [509,510].

### 4.8. Magnetic Therapy

In addition, for magnetic NPs being able to serve as contrast enhancers during MRI (see above), these type of NPs could also be used for therapy, i.e., magnetic therapy (MTH), and thus used as a theranostic platform. Both alternating-current (AC) and direct-current (DC) based magnetic fields could be utilized for this type of technique, although the most commonly used option called magnetic hyperthermia uses AC-based magnetic fields [300]. The Brownian and Neel relaxation processes are the two sources of heat generation during the AC-based magnetic hyperthermia option [511]. The smaller the NP used the more the Neel relaxation process will dominate over the Brownian in contributing to the heat generation in targeted tissue. If instead using a DC-based magnetic field is used the process of magnetocytolysis is utilized in order to induce cellular disruption. 

Regarding PCa and BC related applications using a NP-based MTH technique there are only a few publications available [493,512,513,514,515,516]. For example, Han et al. recently evaluated a theranostic strategy based on Fe_3_O_4_/Au NPs used for prostate-specific antigen detection, MRI, as well as magnetic hyperthermia [512]. For BC, Yao et al. recently investigated a multifunction therapy platform based on silica NP and quantum dots for controlled and targeted drug (doxorubicin) release, NIR-based PTT, and AC-based magnetic hyperthermia in a 4T1 BC model, indicating a significant synergistic therapeutic effect [515]. 

## 5. The Prostate Cancer Case

TNPs might play an important role in the future for the detection, diagnosis, and staging, as well as for the therapy of cancerous diseases at different stages. In order to specifically up-date the reader on the current situation regarding the statistics, diagnosis options, staging, recurrence, metastases, as well as some available treatment options for PCa, a short presentation of this is given below.

### 5.1. Background Statistics

Worldwide, PCa is the second most frequently diagnosed cancer in men, and the fourth most common in both sexes combined. Approximately 1.1 million men were diagnosed with PCa in the world during 2012, which is approximately 15% of all cancers diagnosed in men [517]. Prostate cancer is also the fifth leading cause of death related to cancer in men, with 307,000 deaths worldwide during 2012 [517]. In the USA, PCa is the second leading cause of cancer-related deaths and the second most frequently diagnosed cancer, while in Europe it is number one. The American Cancer Society estimated that 180,890 men would be diagnosed with PCa during 2016 in USA, and about 26,120 would die from the diseases [518]. The International Agency for Research on Cancer (IARC) concluded that during 2012 in Europe close to 345,000 men were diagnosed with PCa. Although more effort has been directed towards early detection through screening, 72,000 men died of PCa in Europe in 2012 [519,520,521,522,523,524,525,526]. Notably, there is less variation in mortality worldwide than is observed for the incidence. This is explained by the PSA testing having greater effect on incidence than on the mortality [517]. The development of improved therapy modalities should therefore be prioritized and targeted therapies based on TNPs are promising candidates to increase the therapeutic efficacy and chance for survival of this category of patients. Several studies of the therapeutic efficacy and toxicity of RIT against PCa have been performed [492,527,528,529,530,531,532,533,534,535,536,537,538,539,540,541,542,543,544].

### 5.2. Diagnosis, Staging, Recurrence, and Metastases

A transrectal ultrasonography—guided pathologic examinational procedure is applied during tumor diagnosis of PCa. The extent of the localized PCa tumor is also estimated by digital rectal examination and PSA testing, sometimes supplemented using CT, bone scanning, or multiparametric MRI [545]. The staging procedure for malignant PCa tumors as outlined in the National Comprehensive Cancer Network (NCCN) guidelines [546], should follow the TNM (Tumor—regional lymph Nodes—Metastasis) classification developed by the Union for International Cancer Control (UICC) and published by the American Joint Committee on Cancer (AJCC), as well as the International Federation of Gynecology and Obstetrics (FIGO), staging manuals [547]. 

Positron emission tomography in combination with CT is used for the staging of lymph-node metastasis involvement. Depending on stage, ^18^F-FDG, ^18^F-choline, or ^11^C PET could be used [548,549,550,551,552,553,554,555,556]. The use of PET in combination with MRI may also help detect PCa as well as improve accuracy of staging [557,558,559,560]. Regarding the estimation if the PCa under investigation is clinically insignificant/indolent (CIPC) or significant/aggressive the Epstein criteria could be used, taken into account its limitations and many variations [561,562,563,564,565,566]. Better criteria deciding CIPC or not CIPC could help minimize the amount of under- and overtreated men having PCa [521]. Regardless whether CIPC or not CIPC, monitoring the disease is usually performed using PSA testing, complemented with MRI and/or PET/CT if PSA level is rising [567]. 

Regarding the treatment of localized PCa radiation therapy (RT) and radical prostectomy (RP) are established protocols, although resulting in up to 50% of PSA recurrence often referred to as biochemical recurrence (BCR) [568]. The PSA doubling time, the Gleason score, and the pathologic T-stage determines the time between BCR and when metastases are confirmed [488,569]. In order to be able to determine if the recurrence is local or in the form of metastases, ^11^C-choline based PET/CT and/or MRI are often used [488,545,551,570,571,572,573]. Regarding metastases, the skeleton and regional lymph nodes are the most common sites, with >80% of the men succumbing to PCa having metastases in the skeleton [574]. Bone scintigraphy, as SPECT in conjunction with CT (SPECT/CT), using Technetium-99 (^99m^Tc)-methylene di-phosphonate is often used to estimate the degree of metastases in the skeleton [567,575]. The use of ^18^F-fluoride PET/CT might also be an option to be used for detecting and classifying metastases in the skeleton [545]. 

## 6. Theranostic Nanoparticles for Prostate and Breast Cancer

For cancer applications in general there is an increasing number of publications regarding multifunctional TNPs, exemplified by a limited selection of references [1,76,576,577,578,579,580,581,582,583,584,585,586,587,588,589,590,591]. For PCa and BC there is only a limited number of publications, some of which already have been mentioned above in conjunction with the presentation of the different type of NPs as well as different imaging and therapy options available for TNPs [1,131,230,345,358,476,477,478,592,593]. As TNPs combine into one nanovehicle both imaging and therapy, the presentation of selected representative examples below illustrate some of these combinations evaluated so far for PCa and BC. 

Lin et al. developed and evaluated a novel multifunctional NP-based platform for simultaneous imaging and therapy of PCa using LNCaP and PC3 cells in a mouse model [476]. The imaging was achieved by using NIR activatable fluorescence NPs enabling optical imaging and therefore real-time monitoring of the drug delivery. The therapy was achieved by simultaneous targeted delivery of heat, ROS, and heat-chock protein 90 (Hsp90) inhibitor. Their porphyrine-based system was able to generate enough heat and ROS simultaneous with light activation in order to achieve a dual PTT/PDT therapy. The developed formulations of Hsp90 inhibitors also enabled a decrease of the level of pro-survival and angiogenic signaling induced by the PTT and PDT treatment, which sensitizes the tumor cells to the phototherapy. The authors concluded that by using their PCa-specific and image-guided minimally invasive NP-based PTT/PDT drug delivery system, in conjunction with the Hsp90 inhibitors, could enhance the therapeutic efficacy for PCa.

In a paper by Vaillant et al. it was investigated the possibility of developing and using a targeting molecule against the cation-independent mannose 6-phosphate receptor (M6PR), over-expressed in especially the LNCaP cell line [477]. The targeting molecule was a mannose 6-phosphate analogue, synthesized in six steps, which was grafted onto functionalized silica NPs. Experiments were performed both in vitro and in vivo using PDT and showed promising results regarding both targeting, imaging, and therapy of PCa. Regarding the developed biomarker and the M6PR investigated, the authors especially emphasize that the target fulfill important characteristics, namely (i) over-expression in 84% of PCa tissues; (ii) no expression in normal tissues or non-cancerous hypertrophy of prostate; and (iii) over-expression in low-grade cancers. Therefore, M6PR is according to the authors a promising target for non-invasive personalized therapy of PCa, with the possibility of future theranostic applications. 

In a paper by Agemy et al. the targeting of the tumor vasculature was investigated for both therapy and imaging of PCa [358]. By screening phage-displayed peptide libraries they identified specific targets in the vessels of PCa tumors. One such peptide, the penta-peptide Cys-Arg-Glu-Lys-Ala, recognizes a fibrin-fibronectin complex located in tumor vasculature. By using SPIONs coated with this peptide in 22Rv1-and LAPC9-PCa cells xenograft mice models an accumulation in tumor vessels was achieved after intravenous injection, which in its turn caused additional clotting and thereby additional sites for the TNPs to bind to in the tumors. No clotting was seen in other parts of the body. Imaging was performed by MRI. The addition of an anti-cancer drug, to these tumor vasculature-blocking TNPs, is hypothesized by the authors to increase the therapeutic efficacy even further. 

In a study by Li et al. a BC xenograft-mice model was used to evaluate the imaging and therapeutic efficacy of self-assembled gemcitabine-Gd(III)-based pegylated 50 nm TNPs [1]. The anti-cancer drug gemcitabine combined with the MRI contrast agent Gd(III) used in this setting for the BC cell line MDA-MB-231 showed a high in vivo antitumor efficacy compared to saline control; median tumor volume equal to 188 and 695 mm^3^ 28 days post injection, respectively. The level of toxicity was indistinguishable compared to controls, drug loading capacity of the TNPs higher than compared to other systems [35,36], and the in vivo MRI-signaling efficacy comparable with other similar NPs [30,594]. 

In vitro experiments were performed by Choi et al. in which they evaluated enzyme-activatable TNPs for NIR-fluorescence imaging and a combination of PDT and CTH of TNBC [478]. The photosensitizer chlorin e6 (Ce6) conjugated to hyaluronic acid (HA) formed Ce6-HA NPs by self-assembly. Then, the anticancer topoisomerase-1 inhibitor camptothecin (CPT) was encapsulated inside these NPs forming the final TNPs. Treatment using the enzyme hyaluronidase induced activation of singlet oxygen generation and NIR fluorescence, as well as the release of CPT from the TNPs. The light irradiation of treated TNBC cells further enhanced the therapeutic efficacy significantly. An up-dated and well written review on the subject of targeted NPs for image-guided treatment of TNBC has been written by Miller-Kleinhenz et al. and discusses, for example, subtypes, biomarkers, and potential surface targets for TNBC [242]. 

Ansari et al. demonstrated in a study the feasibility of a TNP incorporating both tumor specificity, enzyme-activated prodrugs, and in vivo imaging possibilities by conjugating the FDA-approved magnetic iron-oxide NP ferumoxytol to a matrix metalloproteinase-activatable peptide conjugate of the colchicine analogue azademethyl-colchicine [592]. Intravenous injections of the TNPs into MMTV-PyMT (mouse mammary tumor virus-polyoma middle-T-antigen) BC tumor-bearing mice resulted in a significant anti-tumor efficacy compared to controls, with no detectable normal-tissue toxicity, explained by a significant tumor accumulation of the TNPs shown by MRI. The results are important since the MMTV-PyMT cells are considered to be a good model for BC metastasis [595]. It should be noted that by March 30, 2015, FDA changed its prescription instructions for the ferumoxytol-based anemia drug Feraheme^®^ due to risk of serious allergic reactions [596]. 

In conclusion, theranostic NPs applied in an individualized targeted nanomedicine setting have a high potential to become one of the most valuable technologies for the detection, diagnosis, and therapy of PCa and BC. The tumor cell specific multifunctionality of such nanovehicles could enable earlier detection of the diseases, as well as increased sensitivity and specificity during diagnosis. The TNPs also have the potential to increase the likelihood of survival as well as decreasing systemic toxicity for treated patients, compared to the options available today. There are many combinatorial possibilities when constructing TNPs, and all of them have pros and cons as illustrated in this paper. Clinical trials need to be performed in order to give the U.S. Food and Drug Administration, its European Union equivalence European Medicines Agency, and other national medicine-regulatory authorities, the possibility to evaluate relevant TNP options further. This will hopefully add to the list of NP-based drugs under clinical evaluation or already clinically approved, and listed in Table 1 below, also theranostic applications for both PCa and BC.

## Figures and Tables

**Table 1:** Nanoparticle-based drugs for PCa and BC, approved or under clinical evaluation. Listed are also examples of drugs for solid cancers in general, since they also might be applicable to PCa and BC in the future.

Cancer	Specific Indication	Nanoparticle	Drug	Product	Phase	Company
PCa	US enhancement imaging	Phospholipid microbubbles	-	SonoVue®	Phase III	Bracco Diagnostics Inc.
	Metastatic CRPC	Polymeric	Docetaxel	BIND-014 (Accurin™)	Phase II	Pfizer Inc/BIND Therapeutics Inc.
	Hormone refractive PCa	Albumin-based NP	Paclitaxel	Abraxane®	Phase II	Celgene Corporation
	Androgen independant PCa	Liposome	Doxorubicin	Doxil®	Phase II	Janssen Pharmaceuticals Inc.
	-	Iron NP	Iron NP	Magnablate	Phase I/0	University College London Hospitals
BC	Metastatic BC	Liposome	Doxorubicin	Myocet™	Approved	Teva UK Ltd.
	Metastatic BC	Albumin-based NP	Paclitaxel	Abraxane™	Approved	Celgene Corporation
	-	Micelle (polymeric)	Paclitaxel	Genexol-PM™	Approved	Samyang Pharmaceuticals Co.
	US enhancement imaging	Lipid microspheres	-	Definity®	Approved	Lantheus Medical Imaging Inc.
	Metastatic BC	Liposome	Paclitaxel	LIPUSU®	Phase IV	Nanjing Luye Sike Pharmaceutical Co., Ltd.
	Metastatic BC	Polymeric conjugate	Irinotecan	NKTR-102	Phase III	Nektar Therapeutics
	Refractory chest wall BC	Liposome	Doxorubicin	ThermoDox™	Phase II/I	Celsion Co.
	-	Micelle (polymeric)	Paclitaxel	NK105	Phase III	NanoCarrier Co., Ltd.
	Advanced/metastatic BC	HER2-liposome	Doxorubicin	-MM-302	Phase III/II/I	Merrimack Pharmaceuticals Inc.
	Tripple-negative metastatic BC	Liposome	Doxorubicin	Doxil®	Phase II	Janssen Pharmaceuticals Inc.
	-	Liposome	Doxorubicin	Caelyx®	Phase II	Janssen-Cilag Ltd.
	Tripple-negative metastatic BC	Liposome	Paclitaxel	EndoTAG-1	Phase II	MediGene AG
	Metastatic	Liposome	Paclitaxel	LEP-ETU	Phase II	Insys Therapeutics Inc.
	Advanced recurrent/metastatic BC	Liposome	Mitoxantrone	Mitoxantrone HCL Liposome	Phase II	CSPC ZhongQi Pharmaceutical Technology
	Metastatic BC	Micelle (polymeric)	Paclitaxel	Nanoxel™	Phase I	Samyang Pharmaceuticals Co.
	US enhancement imaging	Phospholipid microbubbles	-	SonoVue®	Pilot	Bracco Imaging Inc.
	Metastatic/locally recurrent	Micelle (polymeric)	Paclitaxel	Cynviloq	Not provided	Sorrento Therapeutics Inc.
Solid cancers	Advanced tumors	Liposome	Curcumin	Lipocurc	Phase II/I	SignPath Pharma Inc.
	Advanced tumors	Micelle	Gemcitabine/Cisplatin	NC-6004 Nanoplatin	Phase II/I	NanoCarrier Co., Ltd.
	Advanced tumors	Cyclodextrin-based NP	Docetaxel	CRLX301	Phase II/I	Cerulean Pharma Inc.
	-	Micelle (polymeric)	Docetaxel/Taxotere	Docetaxel-PM DOPNP201	Phase I	Samyang Pharmaceuticals Co.
	-	Micelle	Docetaxel	CriPec	Phase I	Cristal Therapeutics
	Advanced tumors	Micelle (polymeric)	Cisplatin/paclitaxel	NC-4016 DACH-Platin micelle	Phase I	NanoCarrier Co Ltd/MD Anderson Cancer Center
	-	Liposome	Eribulin mesylate	Halaven E7389-LF	Phase I	Eisai Co., Ltd.
	-	Liposome	Mitomycin-C	Promitil®	Phase I	LipoMedix Pharmaceuticals Inc.
	Refractory/recurrent tumors	Liposome	Topotecan, docetaxel, CP	SGT-53	Phase I	SynerGene Therapeutics Inc.
	-	Liposome	RB94 plasmid DNA	SGT-94	Phase I	SynerGene Therapeutics Inc.
	Advanced tumors	Liposome	^188^Re-BMEDA	^188^Re-BMEDA	Phase I	INER, Taiwan
	Advanced tumors	Albumin-based NP	Thiocolchicine	ABI-011	Phase I	NantBioScience Inc.
	-	Lipid	DsiRNA	DCR-MYC	Phase I	Dicerna Pharmaceuticals Inc.
	Advanced recurrent tumors	Liposome	siRNA	siRNA-EphA2-DOPC	Phase I	MD Anderson Cancer Center
	Advanced/refractory tumors	Liposome	Cisplatin	LiPlaCis	Phase I	Oncology Venture/LiPlasome Pharma A/S
	Advanced solid tumors	Polymeric	AZD2811, Irinotecan	AZD2811 (Accurin™)	Phase I	AztraZeneca/BIND Therapeutics Inc.

US = Ultra sound; CRPC = Castration resistant prostate cancer; PSMA = Prostate-specific membrane antigen; AZD2811—Aurora B kinase inhibitor; DsiRNA = Double stranded small interfering RNA; siRNA = small interfering RNA; CP = Cyclophosphamide; NP = Nanoparticle; EndoTag = Endothelial targeting agent; LEP-ETU = liposome entrapped paclitaxel easy-to-us; LIPUSU = Paclitaxel liposome for injection; BMEDA = (2-mercaptoethyl)-*N*’,*N*’-diethylethylenediamine.
